# Development and Validation of RAA-CRISPR/Cas12a-Based Assay for Detecting Porcine Rotavirus

**DOI:** 10.3390/ani14233387

**Published:** 2024-11-25

**Authors:** Siyu Huang, Longhuan Du, Song Liu, Qingcheng Yang, Changwei Lei, Hongning Wang, Liu Yang, Xin Yang

**Affiliations:** 1 Animal Disease Prevention and Food Safety Key Laboratory of Sichuan Province, College of Life Sciences, Sichuan University, Chengdu 610064, China; skye-SCU@outlook.com (S.H.); liusong2355@163.com (S.L.); 18383931853@163.com (Q.Y.); leichangwei@126.com (C.L.); whongning@163.com (H.W.); 2 Animal Breeding and Genetics Key Laboratory of Sichuan Province, Sichuan Animal Science Academy, Chengdu 610066, China; longhuan_du@163.com; 3 National Center of Technology Innovation for Pigs, Chongqing 402460, China; yangliuldz@163.com

**Keywords:** CRISPR/Cas12a, porcine rotavirus, RAA, detection

## Abstract

Piglet diarrhea poses significant economic losses to the pig industry, posing a worldwide challenge that urgently needs to be addressed in pig breeding practices. Porcine rotavirus (PoRV) is an important viral diarrhea pathogen in piglets, with a high incidence rate and a tendency to cause growth retardation. To enhance the sensitivity and specificity of PoRV detection, we sequenced the NSP3 gene of G5 and G9 genotypes of rotavirus A (RVA), enabling simultaneous detection of the two serotypes. Subsequently, we developed a rapid PoRV detection method using a combination of recombinase-aided amplification (RAA) and CRISPR/Cas12a. In this method, Cas12a binds to RAA amplification products, guided by CRISPR-derived RNA (crRNA), which activates its cleavage activity and releases fluorescence by cutting FAM-BHQ-labeled single-stranded DNA (ssDNA). In the optimized reaction system, the recombinant plasmid PoRV can achieve a highly sensitive reaction within 30 min at 37 °C, with a detection limit as low as 2.43 copies/μL, which is ten times higher in sensitivity compared to the qPCR method. Results from specificity testing indicate that no cross-reactivity was observed between the RAA-CRISPR/Cas12a analysis of PoRV and other viral pathogens, including PoRV G3, PoRV G4, porcine epidemic diarrhea virus (PEDV), porcine epidemic diarrhea (PDCoV), and porcine reproductive and respiratory syndrome virus (PRRSV). In the clinical sample detection using the RAA-CRISPR/Cas12a method and qPCR, Cohen’s Kappa value reached as high as 0.952. Furthermore, this approach eliminates the need for large-scale instrumentation, offering a visual result under an ultraviolet lamp through fluorescence signal output.

## Simple Summary

Porcine rotavirus is a significant diarrhea virus in pig farming, particularly causing fatal dehydration in suckling piglets younger than 7 days, thereby resulting in substantial economic losses to the swine industry. In this study, we have established a rapid detection and analysis system for Group A porcine rotavirus using Recombinase Aided Amplification (RAA) combined with Clustered Regularly Interspaced Short Palindromic Repeats (CRISPR) technology for the first time. The recombinant plasmid PoRV achieved a high-sensitivity response within 30 min at 37 °C with a detection limit as low as 2.43 copies/μL, representing a 10-fold higher sensitivity compared to the qPCR method. Specificity testing results showed that the RAA-CRISPR/Cas12a analysis for porcine rotavirus did not react with other common porcine diarrhea viruses. Furthermore, this method allows visualization of results using blue light, making this accurate and portable detection method hold great potential for rotavirus control in pig farming.

## 1. Introduction

Rotavirus (RV), a non-enveloped double-stranded RNA virus belonging to the *Reoviridae* family [1], is the primary cause of viral gastroenteritis in infants and animals worldwide. Posing severe challenges to pig health management, rotavirus is widely distributed and highly resistant to the environment. The disease caused by PoRV is usually self-limiting gastroenteritis, particularly in suckling piglets under 7 days of age, who can suffer fatal dehydration as a result. The impact of PoRV on piglets is typically more severe during outbreaks in intensive farming environments. The most commonly infected pigs belong to rotavirus A (RVA), rotavirus B (RVB), and rotavirus C (RVC) [2]. Currently, for RVA, the Rotavirus Classification Working Group (RCWG) has identified at least 42 G and 58 P genotypes. Rotavirus was first isolated from diarrheal pigs in China in 1982, indicating widespread infection among pigs in our country at that time [3]. Interspecies transmission of RVA among multiple hosts significantly influences viral evolution. Twelve G genotypes and eighteen P genotypes of RVA detected in pigs are associated with pig infections or diseases, among which G3, G5, and G9 remain the dominant genotypes, while G9P[23]c, G9P[7], and G5P[7] are prevalent genotypes in Chinese pigs [4,5]. In recent years, RVA epidemic reports in China have documented a prevalence of approximately 16.65% among piglets, with both clinical diarrhea and asymptomatic cases being reported [5,6].

The diagnosis of pathogens is crucial for disease prevention and control. However, current diagnostic methods such as PCR/qPCR face numerous challenges, including complex operations, high costs, and susceptibility of results to contamination [7]. Since the early 1990s, various isothermal amplification techniques have been developed successively [8,9]. Notably, this technology stands out for its rapidness, simplicity, specificity, and sensitivity without the need for expensive and complex thermal cycling equipment. It surpasses PCR in various aspects, from sample processing to subsequent detection, leading many researchers to believe that isothermal amplification could emerge as a viable alternative to PCR [10,11]. Among these, RAA, as an emerging in vitro amplification technology, breaks through temperature and location constraints, enabling exponential amplification under constant temperature conditions of 37 °C within a short time. Given its simplicity and rapidness, RAA holds great potential in the field of nucleic acid amplification [8,12]. In recent years, the integration of detection with the CRISPR system has pointed to a promising new direction for the development of isothermal amplification [13].

The CRISPR/Cas system operates as an adaptive immune mechanism in bacterial and archaeal systems, empowering bacterial cells to eliminate foreign genetic elements and providing resistance against viral and bacteriophage infections [14]. In 2016, Pardee and his team integrated the CRISPR-Cas9 system with nucleic acid sequence-based amplification (NASBA) to precisely differentiate Zika virus strains in a macaque model, marking the debut of a detection method grounded in CRISPR/Cas technology [15]. Unlike Cas9, which requires both crRNA and tracrRNA for guidance, Cas12a can be directed by a single crRNA alone, without the need for tracrRNA [16]. Among these, CRISPR/Cas12a technology, which harnesses the unique trans-cleavage activity of the Cas12a protein, has broken the limitations of traditional molecular diagnostic technologies and ushered in a new era in the field of in vitro diagnostics (IVD) [16,17]. Furthermore, the incorporation of the original spacer enhances the specificity and complementarity of Cas12a [17,18]. These advantages position the CRISPR-Cas12a system as a novel and promising tool in various bioassay studies [19].

Therefore, this study aims to overcome the limitations of current nucleic acid amplification methods and improve RVA diagnosis by developing an RAA-CRISPR/Cas12a detection system for PoRV. By incorporating two specific crRNAs into the CRISPR system, simultaneous detection of two serotypes of PoRV, namely G5 and G9, is achieved. The entire reaction can be completed within 30 min at 37 °C, with a sensitivity for virus DNA detection reaching a single copy number. Consequently, this platform holds the potential for rapid, accurate, and low-cost point-of-care diagnosis of porcine rotavirus A, offering a swift, simple, and sensitive tool for PoRV outbreak surveillance.

## 2. Materials and Methods

### 2.1. Plasmid, Virus Strain, and Genome

The competent DH5α cells utilized in this experiment were procured from Shanghai Pudi Biotechnology Co., Ltd. (Shanghai, China). The PMD-19T plasmid vector was obtained from TaKaRa (Kyoto, Japan). Two specific NSP3 gene fragments were synthesized, namely NSP3 from PoRV (G3, MK597968.1) and NSP3 from PoRV (G4, MT784854.1), all synthesized by Tsingke Biotech Co., Ltd. (Beijing, China). Additionally, all strains involved in this study, including the porcine epidemic diarrhea virus (CV777), porcine reproductive and respiratory syndrome virus (SCcd17), and porcine deltacoronavirus (SC), were maintained within our laboratory.

### 2.2. Primer Probe and crRNA Design

To identify the most conserved region within the target gene sequence of PoRV, qPCR primers were designed using SnapGene (Version 3.2.1).

We downloaded about 600 full-genome G5 and G9 RVA sequences from NCBI and aligned their gene reference sequences using Mega7 software. Subsequently, conserved sites on the NSP3 gene were selected to design two crRNAs (PoRV-crRNA1, PoRV-crRNA2). The designed crRNAs were reverse complemented and subsequently appended with the hairpin structure of the LbCas12a protein.

The synthesis of crRNA involves multiple steps, including in vitro transcription and purification, which are carried out according to the protocols provided by the T7 High Efficiency In Vitro Transcription Kit and the Centrifugal Column-based RNA Purification and Concentration Kit from Sangon Biotech (Shanghai, China), respectively, and stored at −80 °C.

As indicated in Table 1, the ssDNA probes used for fluorescence signal output possess distinct modifications at their termini, preventing non-specific cleavage by LbCas12a. To amplify the target sequence fragment, RAA primers were designed to adhere to established principles. For RVA, the RAA primers were designed using Primer 5 software and are listed in Table 1. All primers, genomic DNA (gDNA), and probe sequences were synthesized by Sangon Biotech (Shanghai, China).

### 2.3. Preparation of PoRV Standard Plasmid

The NSP3 genes of the G5 and G9 genotypes of RVA exhibit a relatively high degree of conservation. Upon alignment using MEGA (Version 7), it was uncovered that all sequences shared 15 distinct partial single-nucleotide polymorphisms, which are detailed in Supplementary Table S1. Synthetic NSP3 fragments targeting PoRV1, PoRV4, PoRV10, and PoRV12, along with NSP3 gene fragments from PoRV(G3) and PoRV(G4), were used as templates. The primers listed in Table 1 were employed for the preparation of recombinant plasmids. These target sequences were amplified using Takara’s high-fidelity enzyme, PrimeSTAR^®^ Max DNA Polymerase, and subsequently cloned into the plasmid vector pMD19-T (Takara, Kyoto, Japan).

The resulting recombinant plasmids, designated as pMD19-PoRV1, pMD19-PoRV4, pMD19-PoRV10, and pMD19-PoRV12, were individually transformed into *Escherichia coli* DH5α cells (Vazyme, Nanjing, China). Plasmid extraction was carried out using a mini-plasmid kit (TIANGEN, Beijing, China). The concentrations of the extracted plasmids were measured utilizing a Nanodrop 2000 spectrophotometer (Thermo Fisher Scientific, Waltham, MA, USA) and stored at −20 °C for future use.

### 2.4. The Principle of RAA-CRISPR/Cas12a for Detection of PoRV

The flow diagram of the RAA-CRISPR/Cas12a assay developed in this study for the detection of PoRV is presented in Supplementary Figure S1a [20,21]. The RNA in the sample is reverse transcribed into cDNA, followed by an RAA-CRISPR/Cas12a reaction. The final result of the reaction can be observed using a UV transilluminator or quantified with a qPCR instrument to determine specific fluorescence values [21]. Moreover, the detailed principle of RAA and Cas12a is illustrated in Supplementary Figure S1b.

Since crRNA permits mutations on the target sequence of no more than two single nucleotides, two crRNAs, crRNA1 and crRNA2, were designed for the unique variability of the PoRV target in this study [22,23]. As depicted in Supplementary Figure S1c, crRNA1, and crRNA2 can direct Cas12a to recognize the four typical mutation types present in the designed standards (PoRV1, PoRV4, PoRV10, and PoRV12). By incorporating both crRNA1 and crRNA2 into the Cas system, the identification of G5 and G9 PoRV becomes feasible.

### 2.5. Establishment of an RAA-CRISPR/Cas12a Assay

According to the manufacturer’s protocol (ZC BIOSCIENCE, Hangzhou, China), the RAA reaction system is comprised of Buffer A, Buffer B, forward and reverse primers, and target nucleic acid. Prior to the reaction, an RAA premix is prepared by combining 25 μL of Buffer A, 13.5 μL of double-distilled water (ddH_2_O), 2 μL of forward primer (10 μM), and 2 μL of reverse primer (10 μM) into a tube containing the lyophilized powder. This mixture is thoroughly agitated until uniform. To significantly reduce costs compared to the 50 μL reaction system recommended in the RAA kit, this experiment scales down the reaction volume to 10 μL. Specifically, 8.5 μL of the premix is aliquoted, followed by the addition of 1 μL of target nucleic acid and 0.5 μL of Buffer B, which acts as the activator for the entire reaction. Therefore, it is essential to immediately place the mixture at 37 °C upon the addition of Buffer B.

According to the manufacturer’s protocol (Tolo Biotech, Shanghai, China), the CRISPR/Cas12a reaction system contains 2 μL of LbCas12a, 2 μL of crRNA (composed of 1 μL of crRNA1 and 1 μL of crRNA2), 2 μL of Buffer 3, 0.5 μL of RNA Inhibitor (RRI), 1 μL of reporter ssDNA (FQ-Reporter), 9 μL of ddH_2_O, and 4 μL of the RAA reaction product. The entire system is incubated at a constant temperature of 37 °C. Optionally, nucleic acid eradicants can be used to mitigate the risk of aerosol contamination. The reaction is then carried out in a qPCR instrument for 10 min, during which fluorescence signals are collected every minute through the FAM channel.

### 2.6. Optimization of RAA-CRISPR/Cas12a Reaction Conditions

In this experiment, the reaction temperature and time for RAA, along with the concentrations of Cas12a and crRNA and the amount of FQ-reporter used in the Cas12a assay, were all optimized. According to the instructions of the RAA detection kit, RAA can maintain high specificity and high product concentration with a reaction time of 30 min and a reaction temperature of 39 °C. Considering that DNA polymerase affects the amplification efficiency of the RAA reaction, we sequentially set the reaction temperatures to 37 °C, 38 °C, 39 °C, 40 °C, and 41 °C. Additionally, since prolonged reactions can lead to non-specific amplification in RAA, this study also optimized the reaction time of RAA. To determine the optimal reaction time for RAA, five different reaction times (10, 15, 20, 25, and 30 min) were designed.

The CRISPR/Cas12a system is utilized for the recognition and cleavage of RAA products. Due to the numerous influencing factors involved in the Cas system, it is necessary to optimize the Cas12a reaction conditions. By comparing the fluorescence intensity at different reaction time points, the optimal reaction time for the Cas12a reaction can be selected. Subsequently, we optimized the concentrations of Cas12a (ranging from 0.01 to 1 μM) and crRNA (ranging from 10 to 500 nM). Finally, the amount of FQ-reporter in the Cas system was adjusted within an optimization range of 0.5 μL to 2 μL.

### 2.7. Exploration of the Detection Capability of RAA-CRISPR/Cas12a

#### 2.7.1. Feasibility Test

Based on the target-conserved sequences of PoRV, multiple RAA primers and crRNAs were designed to screen out the optimal experimental combination for the RAA-CRISPR/Cas12a assay.

#### 2.7.2. Comparison of the Sensitivity Between RAA-CRISPR/Cas12a and qPCR

The concentration of the PoRV standard (plasmid) was determined, and the copy numbers were calculated using a corresponding formula. The plasmid was then subjected to a tenfold gradient dilution to obtain test samples ranging from 10^4^~10^0^ copies/μL. The RAA reaction was performed on the test samples following the steps outlined in Section 2.5, with ddH_2_O serving as the negative control. After completion, the samples were observed under a UV lamp to determine the limit of detection (LOD) of the RAA-CRISPR/Cas12a assay. The fluorescence detection of the different gradient samples was conducted in a fluorescence quantitative qPCR instrument using the optimal reaction system determined previously (see Table 2). The thermal cycling protocol was as follows: initial denaturation for 30 s at 95 °C, followed by 40 cycles of 5 s at 95 °C and 30 s at 60 °C. The fluorescence intensities/signals were then detected at the end of each extension step at 60 °C using the QuantStudio 3 Real-Time PCR System (Applied Biosystems, Waltham, MA, USA).

#### 2.7.3. Specificity of RAA-CRISPR/Cas12a for Detection of PoRV

In the specificity assessment, we primarily tested common infectious pathogens in pig farms, such as PoRV, PEDV, and PDCoV. The purpose was to identify whether the detection method established in this study had cross-reactivity with other common pig diseases. RNA from porcine epidemic diarrhea virus (CV777), porcine reproductive and respiratory syndrome virus (SCcd17), and porcine deltacoronavirus (SC), which were preserved in our laboratory, was extracted and reverse-transcribed into cDNA. The RAA reaction was performed on the major pathogens following the steps outlined in Section 2.5, with ddH_2_O serving as the negative control. The optimal reaction system determined previously was used to conduct target-specific fluorescence detection in a fluorescence quantitative qPCR instrument.

#### 2.7.4. Assay Reproducibility of RAA-CRISPR/Cas12a for Detection of PoRV

To determine the assay reproducibility of the method established in this study, we used a standard with 104 copies/μL of PoRV1 as the target and ddH_2_O as the negative control. For each of the different time points, the detection was repeated three times with three replicate groups, utilizing the known optimal detection system.

#### 2.7.5. Detection of Clinical Specimens

A total of 396 clinical samples (including feces and intestinal samples) from diarrheal newborn piglets were collected from 29 pig farms in southwest China (see Supplementary Table S2). Total RNA is extracted from these samples, reverse transcribed into cDNA, and used as the template for qPCR and RAA-CRISPR/Cas12a detection.

## 3. Results and Analysis

### 3.1. Screening of RAA-CRISPR/Cas12a Detection Primers and crRNA

**Figure 1:**
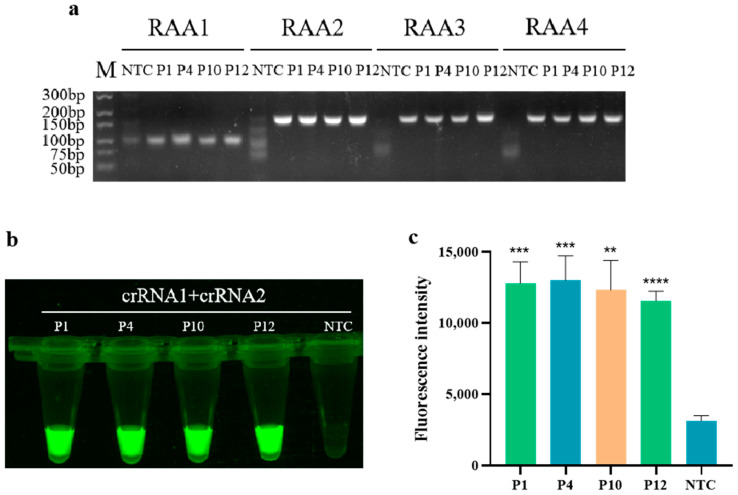
Screening of primers for RAA and CRISPR/Cas12a detecting PoRV. (**a**) Gel electrophoresis of RAA primer screening (M: marker; P1–P4: Four different mutation types of PoRV plasmids designed); (**b**) Fluorescence images for feasibility assessment; (**c**) Fluorescent results of four different PoRV plasmids detected by the RAA-CRISPR/Cas12a assay were collected at 10 min for the Cas12a reaction. Three replicates were conducted for each test. Fluorescence intensity values are shown in the graph as mean ± SD (NTC: negative control, ddH_2_O; ** *p* < 0.01, *** *p* < 0.001, **** *p* < 0.0001).

As shown in Figure 1a, the amplification products of the four pairs of RAA primers were detected by 1% gel electrophoresis. It can be observed that a vague smear band appears in the control group, which is attributed to the presence of various enzymes in the RAA reaction system. Therefore, only a clear and bright band indicates a positive amplification result. The results indicate that all four designed RAA primers can amplify the target sequences of four standards: PoRV1, PoRV4, PoRV10, and PoRV12. Among them, the band of primer RAA2 is the brightest, indicating the highest amplification efficiency in the RAA reaction. Subsequently, primer RAA2 was selected as the optimal primer for the RAA reaction, and both crRNA1 and crRNA2 were targeted to its amplification product. In Figure 1b,c, strong fluorescence is displayed by the four standard plasmids of PoRV1, PoRV4, PoRV10, and PoRV12, confirming the feasibility of the RAA-CRISPR/Cas12a detection method for PoRV. Therefore, PoRV-RAA-F2, PoRV-RAA-R2, crRNA1, and crRNA2 were selected as the optimal combination for subsequent research on PoRV detection.

### 3.2. Optimization Results of the Reaction System

**Figure 2:**
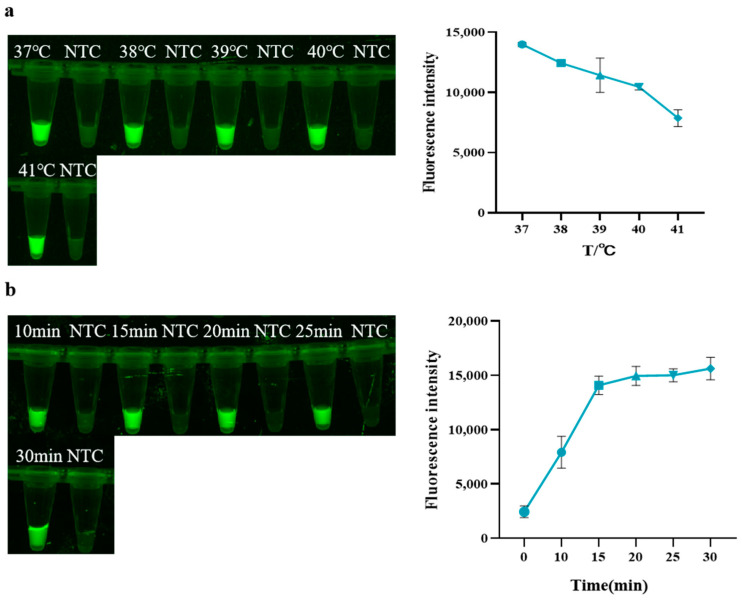
Optimization of RAA. (**a**) Temperature of RAA; (**b**) Time of RAA (NTC: negative control, ddH_2_O).

As shown in Figure 2a, within a temperature range of 37 °C to 41 °C, the fluorescence intensity of the reaction gradually decreases as the temperature increases, indicating a gradual decrease in the concentration of amplification products. Thus, 37 °C was selected as the optimal reaction temperature for RAA. As shown in Figure 2b, when the RAA reaction proceeds for 15 min, the fluorescence intensity upon adding the Cas system gradually reaches a plateau. Therefore, 15 min was chosen as the optimal reaction time for RAA.

**Figure 3:**
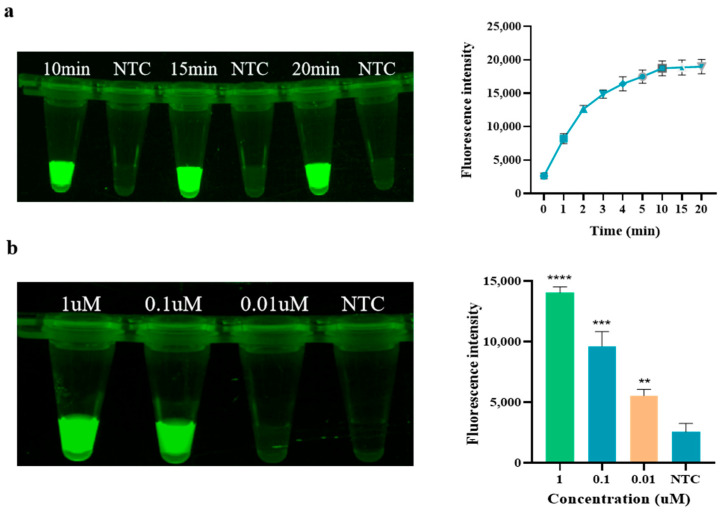

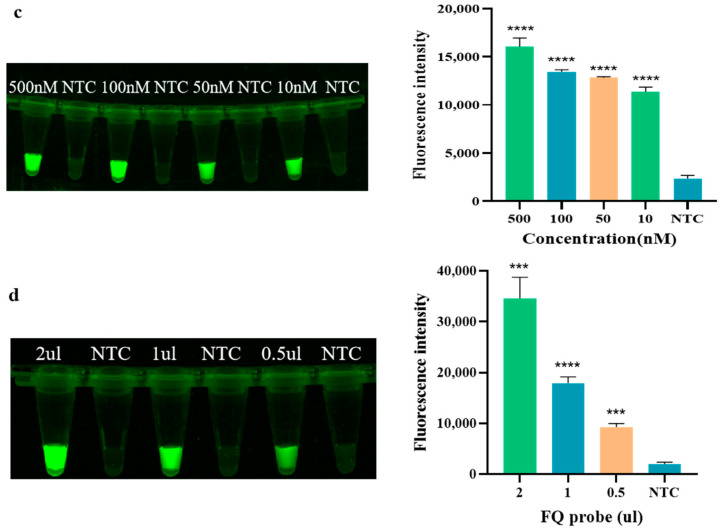
Optimization of CRISPR/Cas12a. (**a**) Time of Cas12a; (**b**) Concentration of Cas12a; (**c**) Concentration of crRNA; (**d**) Dosage of FQ-ssDNA (NTC: negative control, ddH_2_O; **** *p* < 0.0001; *** *p* < 0.001; ** *p* < 0.01).

As shown in Figure 3a, the fluorescence intensity gradually reaches a peak at 10 min of the Cas12a reaction and then remains relatively stable, so the endpoint fluorescence intensity at 10 min of the Cas12a reaction was chosen to measure the Cas12a cleavage level. As the Cas12a concentration increases, the fluorescence intensity gradually increases. The optimal concentration of Cas12a screened in this experiment is 0.1 μM (see Figure 3b), at which point the fluorescence intensity meets the experimental requirements while ensuring the optimal Cas12a content in the reaction system. In the optimization of crRNA concentration, as depicted in Figure 3c, the fluorescence intensity initially increases with the increase in concentration. However, at higher concentrations, such as 500 nM, the fluorescence intensity of the negative control also correspondingly increases, which may compromise the accuracy of subsequent experiments. Therefore, 100 nM of crRNA, which exhibits a high fluorescence intensity without any background signal, was selected for use in the Cas system. Regarding the optimization of the reporter FQ-ssDNA, as shown in Figure 3d, the fluorescence intensity of the reaction increases with the addition of FQ-ssDNA. When the amount of FQ-ssDNA reaches 1 μL, the fluorescence intensity is already sufficient to meet the experimental requirements. Furthermore, compared to using 2 μL, the cost is lower. Therefore, 1 μL of FQ-ssDNA (10 μg/μL) was chosen for use in the Cas system. In summary, the optimal reaction temperature for RAA is 37 °C, and the optimal reaction time is 15 min. In the CRISPR/Cas12a experiment, the optimal reaction time is 10 min, with Cas12a and crRNA concentrations of 0.1 μM and 100 nM, respectively, and the amount of reporter FQ-ssDNA used in the Cas system is 1 μL.

### 3.3. Results of the Specificity Test

**Figure 4:**
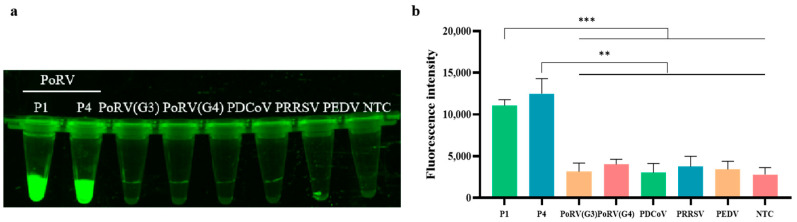
Specificity of RAA combined with Cas12a detecting PoRV. (**a**) Fluorescence image of the Cas12a assay (P1 and P4: Two different base mutant plasmids were designed); (**b**) fluorescent results of different porcine pathogens detected by the RAA-CRISPR/Cas12a assay were collected at 10 min for the Cas12a reaction. Three replicates were conducted for each test. Fluorescence intensity values are shown in the graph as mean ± SD. (NTC: negative control, ddH_2_O; *** *p* < 0.001; ** *p* < 0.01).

The established RAA-CRISPR/Cas12a detection system for PoRV was used to test the nucleic acid recombinant plasmids of G3 and G4 genotypes of PoRV, PEDV, PDCoV, and PRRSV. The results, as shown in Figure 4, indicate that only two different mutant G5 and G9 genotypes of PoRV exhibited high fluorescence intensity, while no fluorescence was detected in other viral samples, including the G3 and G4 genotypes of PoRV. The fluorescence image (see Figure 4a) also shows the same results. This suggests that the RAA primers and the CRISPR/Cas12a system exhibit high specificity for detecting the G5 and G9 genotypes of PoRV. Therefore, the RAA-CRISPR/Cas12a-based PoRV detection method developed in this study demonstrates excellent specificity for the G5 and G9 genotypes of PoRV and can be utilized for the identification and testing of these specific virus types.

### 3.4. Results of the Sensitivity Test Comparing RAA-CRISPR/Cas12a and qPCR

**Figure 5:**
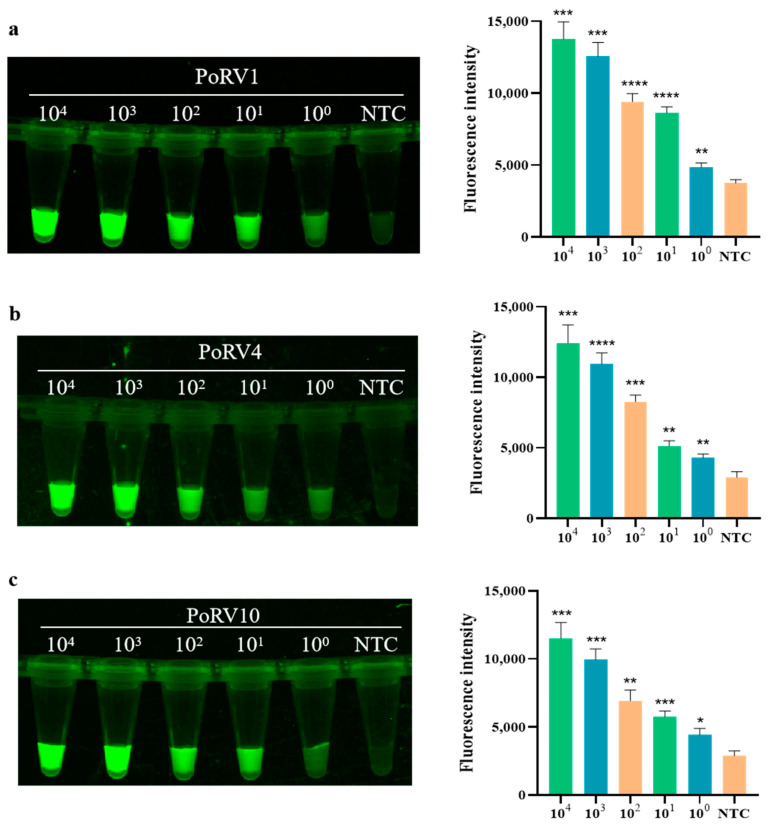

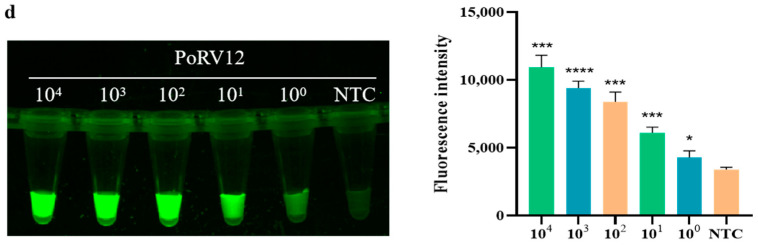
Sensitivity of RAA combined with Cas12a for detecting four standard PoRV plasmids. (**a**) Sensitivity of PoRV1 standard. The fluorescence intensity of each sample was collected at 10 min for the Cas12a reaction; bar graphs represent fluorescent signals for the Cas12a reaction from the fluorescence image, and three replicates were conducted for each test. Fluorescence intensity values are shown in the graph as mean ± SD (NTC: negative control, ddH_2_O; * *p* < 0.05, ** *p* < 0.01, *** *p* < 0.001, **** *p* < 0.0001); (**b**) sensitivity of PoRV4 standard; (**c**) sensitivity of PoRV10 standard; (**d**) sensitivity of PoRV12 standard.

Using the optimal conditions determined in Section 2.7.2, the sensitivity of the detection system was tested. As shown in the bar graph in Figure 5, the fluorescence intensity of the detection increases as the target concentration increases from 10^0^ copies/μL to 10^4^ copies/μL. When the PoRV concentration is greater than 10^3^ copies/μL, the fluorescence intensity increases significantly, designated as strongly positive. When the concentration is between 10^0^ copies/μL and 10^2^ copies/μL, the fluorescence intensity increases linearly, classified as weakly positive. These results indicate that the actual detection limit of the real-time fluorescence signal for PoRV is as low as 2.43 × 10^0^ copies/μL. In the fluorescence graph in Figure 5, the images of the four standards all show a decrease in fluorescence brightness with decreasing concentration, demonstrating the high sensitivity of RAA combined with CRISPR/Cas12a for detecting PoRV.

**Figure 6:**
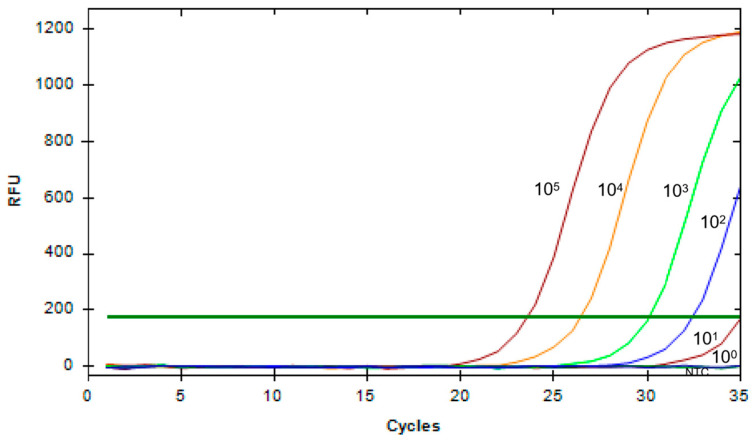
Sensitivity of qPCR detection for PoRV. The PoRV1 standard sample was serially diluted in a 10-fold gradient from 10^5^ copies/μL to 10^0^ copies/μL, and PoRV1 continuous dilutions were amplified using a specific primer set. Concentration unit: copies/μL (NTC: negative control, ddH_2_O).

Simultaneously, a standard method, qPCR, was used for comparison. As shown in Figure 6, as the substrate concentration decreases, the Cq value of the amplification curve gradually increases. When the substrate concentration reaches as low as 10^1^ copies/μL, the fluorescence signal is weakest, thus determining the LOD of qPCR to be as low as 2.43 × 10^1^ copies/μL. In comparison, the sensitivity of the method combining RAA with CRISPR/Cas12a is 10 times higher than that of qPCR. In summary, the combination of RAA and Cas12a is a rapid and highly sensitive tool for detecting G9 and G5 PoRV.

### 3.5. Results of the Assay Reproducibility Test

**Figure 7:**
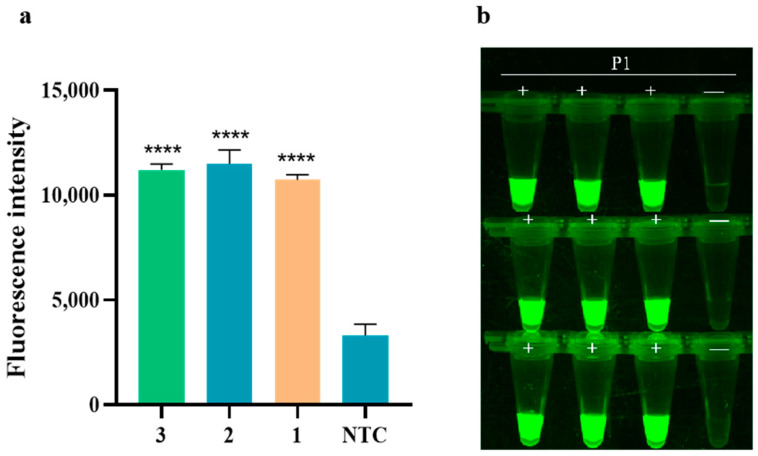
The assay reproducibility of RAA-CRISPR/Cas12a detecting PoRV. (**a**) The fluorescence intensity of PoRV1 was detected by RAA-CRISPR/Cas12a at 10 min, and the method was replicated in triplicate at the same PoRV target concentration. Fluorescence intensity values are shown in the plot as mean ± SD (NTC: negative control, ddH_2_O; **** *p* < 0.0001); (**b**) fluorescence image of PoRV assay reproducibility (P1: PoRV1 standard; +: positive sample; —: negative control).

The study on the assay reproducibility of RAA-CRISPR/Cas12a for detecting PoRV is presented in Figure 7. The results revealed that the fluorescence intensities obtained from the three Cas12a real-time fluorescence assays at 10 min were consistently similar, with no fluorescence signal detected in any of the negative controls. This indicates that the experimental method used for detecting PoRV demonstrates good assay reproducibility.

### 3.6. Clinical Sample Detection Results of RAA-CRISPR/Cas12a and qPCR

**Figure 8:**
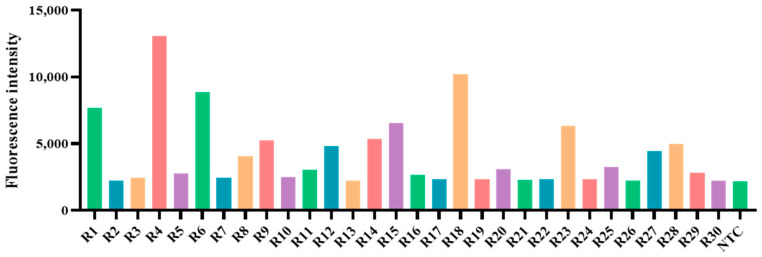
The RAA/Cas12a results of PoRV in some diarrhea samples. The bar chart displays the fluorescent signals detected by RAA-CRISPR/Cas12a for some PoRV samples at the 10-min point of the Cas12a reaction. (NTC: Negative control, ddH_2_O; R1–R30: the number of partial detection samples).

**Figure 9:**
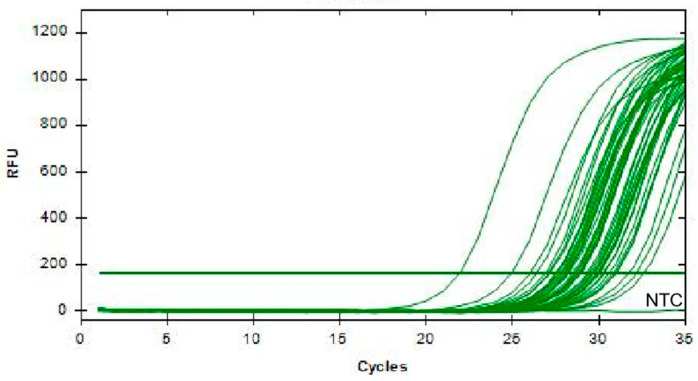
qPCR detection results of PoRV in some samples. (NTC: negative control, ddH_2_O).

A total of 396 clinical specimens were tested using two methods, RAA-CRISPR/Cas12a and qPCR, to determine their infection status with PoRV. The results revealed that RAA-CRISPR/Cas12a identified 54 positive samples (Figure 8), and qPCR detected 50 positive samples (Figure 9), with positive rates of 13.6% (54/396) and 12.6% (50/396), respectively (see Table 3). In summary, with a Cohen’s Kappa value of 0.952, the results of RAA-CRISPR/Cas12a and qPCR in detecting clinical samples are nearly identical, indicating the reliability of RAA-CRISPR/Cas12a in detecting PoRV.

## 4. Discussion

Porcine viral diarrhea is a common clinical disease that can result in high mortality rates among piglets [24]. Among them, PoRV is a significant diarrheal virus in piglets [25]. In recent years, the incidence of mutations in the G and P genotypes of porcine rotavirus A has been increasing worldwide, causing significant economic losses to the livestock industry [24,26]. More importantly, the clinical detection rate of PoRV G9 type has been increasing annually, indicating that the prevalent strain of PoRV is gradually evolving from G5 type to G9 type [27]. This also indirectly reflects that the trivalent attenuated vaccine against RV-TGEV-PEDV (G5 type) has gradually begun to lose its effectiveness [28,29]. Therefore, effective pathogen detection is crucial for the prevention and control of infectious diseases. Commonly used pathogen detection methods include ELISA, PCR, qPCR, and loop-mediated isothermal amplification (LAMP), among others. While ELISA relies on antibody sensitivity, its lengthy incubation time makes it unsuitable for rapid testing [30]. RT-qPCR, on the other hand, offers high sensitivity and is widely recognized as the gold standard for pathogen detection, but it necessitates thermal cycling and complex equipment [31]. Lingyi Wu et al. [32] combined the CRISPR/Cas9 method with electrochemiluminescence, achieving high sensitivity capable of detecting 38 CFU/mL of Listeria monocytogenes genomic DNA, yet the incubation process alone took an hour. In comparison, Shi et al. [31] established a LAMP-based SPIR-12a isothermal method for Shigella detection, which requires a reaction temperature of 60 °C and involves designing multiple primers. Consequently, there is a need to develop a rapid and simple detection method for PoRV. In contrast, the RAA-CRISPR/Cas12a method proposed in this study can be conducted at an ambient temperature of 37 °C, which significantly enhances its efficiency and simplicity.

To better prevent and control PoRV, this study has developed an RAA-CRISPR/Cas12a-based detection method for G5 and G9 genotypes. PoRV is a double-stranded RNA (dsRNA) virus with a highly immunogenic VP6 protein often targeted for detection [28]. However, studies have found that the VP6 gene sequence is prone to rapid mutations, affecting the accuracy of existing detection methods [33,34]. By comparing many PoRV full-genome sequences, we found a conserved NSP3 fragment in G5 and G9 genotypes involved in viral cell entry and mRNA translation that the Cas12a system can recognize [35,36]. Previous studies have shown that Cas12a requires crRNA containing a specific spacer sequence to guide the effector module to the target sequence, thereby activating its specific dsDNA cleavage and non-specific ssDNA trans-cleavage [20,36]. Therefore, based on the unique conservation of PoRV sequences, this study designed two crRNAs: crRNA1 for recognizing normal sequences and one-base mutation sequences, and crRNA2, which recognizes one-base mutation and two-base mutation sequences. Additionally, four plasmids, namely PoRV1, PoRV4, PoRV10, and PoRV12, were designed on the standard template, each containing all types of nucleotide mutations in the target fragment of the NSP3 gene, including two base mutations at common sites and base mutations at PAM sites. In other words, crRNA1 is able to recognize PoRV1, PoRV10, and PoRV12, whereas crRNA2 can recognize PoRV4, PoRV10, and PoRV12. Consequently, when equal amounts of crRNA1 and crRNA2 were added to the Cas system, the results showed that all four positive plasmids produced significant fluorescence, demonstrating the feasibility of this experiment.

During the scientific exploration of RAA-CRISPR/Cas12a’s detection capabilities, a comparison between RAA-CRISPR/Cas12a and qPCR revealed that RAA-CRISPR/Cas12a exhibited numerous detection advantages. To ensure the accuracy and effectiveness of this method’s sensitivity, we investigated the LOD for four plasmids separately. The results indicated that the LOD for rotavirus A by RAA-CRISPR was 10^0^ copies/µL, which was 10 times higher than the LOD of qPCR, confirming the high sensitivity of the method. This was attributed to the high amplification efficiency of RAA and the secondary signal amplification capability of Cas12a. Additionally, the extreme conservation requirement of gene sequences during the primer design phase when integrating RAA with the Cas12a system could also contribute to its low detection threshold [37]. Secondly, the RAA-CRISPR/Cas12a reaction can be completed in 30 min at 37 °C, a temperature readily provided by a constant temperature incubator or water bath. In contrast, qPCR requires complex instruments and a longer duration. Finally, when applying RAA-CRISPR/Cas12a to detect clinical diarrhea samples, compared to the traditional qPCR method, the Cohen’s Kappa value reached as high as 0.952, further validating its authenticity and effectiveness. Despite the numerous advantages of the RAA and CRISPR/Cas12a combination for pathogen detection mentioned above, the limitations of the CRISPR system include the necessity of crRNA for guiding the cleavage of DNA target nucleic acids and its sensitivity to high temperatures.

Overall, using the optimized RAA-CRISPR/Cas12a system, we achieved rapid and sensitive detection of PoRV, enabling visual detection under UV light, with amplification and detection efficiency superior to qPCR. Porcine diarrhea has always been a challenge in pig farming, making the early detection of PoRV particularly crucial [38,39]. This method, characterized by short detection time, high sensitivity, and low cost, enhances applicability.

## 5. Conclusions

This study established a method based on RAA and CRISPR/Cas12a, specifically targeting the prevalent G5 and G9 genotypes of rotavirus A, and represents the first application of integrating RAA with the CRISPR system for the detection of PoRV. RAA achieves rapid nucleic acid amplification in a single step within 15 min, completing the first stage of signal amplification. Additionally, CRISPR/Cas12a exhibits high specificity and sensitivity, enabling secondary signal amplification. Furthermore, CRISPR/Cas12a offers a fluorescent signal output mode, achieving a fluorescent sensitivity of 10^0^ copies/μL within 10 min, surpassing the sensitivity of methods such as qPCR. This method is simple and effective, not only providing a practical tool for PoRV detection but also paving the way for the detection of other prevalent viruses. In the near future, efforts will be made to attain a comprehensive approach to standardizing, normalizing, and simplifying the process, particularly by developing a one-step detection scheme using RAA-Cas12a, with the aim of enhancing the scheme’s efficiency and practicality.

## Figures and Tables

**Table 1:** qPCR primers, crRNA, and RAA primers for PoRV detection.

Name	Primer Sequences (5′→3′)
PoRV-NSP3-F1	ATGGAGTCTACTCAGCAGATGG
PoRV-NSP3-R1	ACCAGAATCATCCATTAC
PoRV-crRNA1	AAUUUCUACUAAGUGUAGAUGAAGCUGCAGUUGUUGCUGC
PoRV-crRNA2	AAUUUCUACUAAGUGUAGAUGAGGCUGCGGUUGUUGCUGC
FQ-reporter	6-FAM-TTTATTT-BHQ1
PoRV-RAA-F1	ATGGAGTCTACTCAGCAGATGGTAAGCTC
PoRV-RAA-R1	TGAATACCCATTAATTCTAATGTTGAAGTG
PoRV-RAA-F2	ATGGAGTCTACTCAGCAGATGGTAAGCTCTATTAT
PoRV-RAA-R2	ACACCAGAGTCATCCATTACATAATCAAATTTAC
PoRV-RAA-F3	GGAGTCTACTCAGCAGATGGTAAGCTCTATTATTA
PoRV-RAA-R3	CACCAGAGTCATCCATTACATAATC
PoRV-RAA-F4	CGAGTCTACTCAGCAGATGGTAAGCTCT
PoRV-RAA-R4	CACCAGAGTCATCCATTACATAATCAAATTTACTT

**Table 2:** qPCR Reaction System.

Name of Component	Dosage
Template plasmid	1 μL
Upstream and downstream primers (10 pM)	1 μL
2X SYBR Green Pro Taq HS Premix (Takara, Kyoto, Japan)	10 μL
RNase-free water	To 20 μL

**Table 3:** Detection results of clinical samples in RAA-CRISPR/Cas12a detection and qPCR assays.

		Test Evaluated (RAA-CRISPR/Cas12a)		
		Positive	Negative	Total
Gold Standard (qPCR)	Positive	50	0	50
	Negative	4	342	346
	Total	54	342	396

## Data Availability

The data are available upon request from the corresponding authors.

## Supplementary Materials

The following supporting information can be downloaded at: https://www.mdpi.com/article/10.3390/ani14233387/s1, Figure S1: The principle of RAA combining CRISPR/Cas12a For detecting porcine diarrhea-related viruses. (a) Principle of Cas12a for detecting G5 and G9 type PoRVA; (b) Detection Process Chart; (c) Principle of RAA and Cas12a; Table S1: Summary of NSP3 gene base mutations in PoRV; Table S2: Information of diarrhea samples.
