# Supplementary material for: Development and Validation of RAA-CRISPR/Cas12a-Based Assay for Detecting Porcine Rotavirus
Source: Animals (Basel). 2024 Nov 25;14(23):3387. doi: 10.3390/ani14233387 (PMC11640193; doi:10.3390/ani14233387)
Supplement: Supplementary file 1 [file animals-14-03387-s001.zip › animals-3261404-supplementary.pdf]

Supplementary Table S1. Summary of NSP3 gene base mutations in PoRV.

Bases / Serial numbers	1	2	3	4	5	6	7	8	9	10	11	12	13	14	15	16	17	18	19	20	21	22	23	24	The number of mutated bases	CrRNA
1	T	T	T	T	G	A	A	G	C	T	G	T	A	G	T	T	G	T	T	G	C	T	G	C	0	1
2	T	T	T	T	G	A	A	G	C	T	G	T	G	G	T	T	G	T	T	G	C	T	G	C	1	1
3	T	T	T	T	G	A	G	G	C	T	G	T	A	G	T	T	G	T	T	G	C	T	G	C	1	1
4	T	T	T	T	G	A	G	G	C	T	G	T	A	G	T	T	G	T	T	G	C	T	G	C	2	2
5	T	T	T	T	G	A	A	G	C	T	G	T	A	G	T	T	G	T	C	G	C	T	G	C	1	1
6	T	T	T	T	G	A	A	G	C	C	G	T	A	G	T	T	G	T	T	G	C	T	G	C	1	1
7	T	T	T	T	G	A	A	G	C	A	G	T	A	G	T	T	G	T	T	G	C	T	G	C	1	1
8	T	T	T	T	G	A	A	G	C	T	G	T	A	G	T	C	G	T	T	G	C	T	G	C	1	1
9	T	T	T	T	G	A	A	G	C	T	G	T	A	G	T	T	G	T	T	G	C	T	T	C	1	1
10	C	T	T	T	G	A	A	G	C	T	G	T	A	G	T	T	G	T	T	G	C	T	G	C	1	1
11	T	T	T	T	G	A	A	G	C	T	G	T	A	G	T	T	G	T	A	G	C	T	G	C	1	1
12	T	T	T	C	G	A	A	G	C	T	G	T	A	G	T	T	G	T	T	G	C	T	G	C	1	1
13	A	T	T	T	G	A	A	G	C	T	G	T	A	G	T	T	G	T	T	G	C	T	G	C	1	1
14	T	T	T	T	G	A	A	G	C	T	G	T	A	G	T	T	G	T	T	G	C	C	G	C	1	1
15	T	T	T	T	G	A	A	G	C	T	G	T	A	G	T	T	G	G	T	G	C	T	G	C	1	1

Supplementary Table S2. Information of diarrhea samples.

Province	City	Number of pig farms with diarrhea	Number of diarrhea samples
Sichuan	Qionglai	6	74
	Suining	1	22
	Guangyuan	1	10
	Nanchong	2	13
	Yibin	4	65
	Neijiang	1	8
	Yaan	5	65
	Bazhong	1	21
Guizhou	Bijie	2	40
	Guiyang	1	18
	Zunyi	1	16
Yunnan	Qujing	1	9
	Kunming	1	9
	Zhaotong	2	26
Total	14	29	396

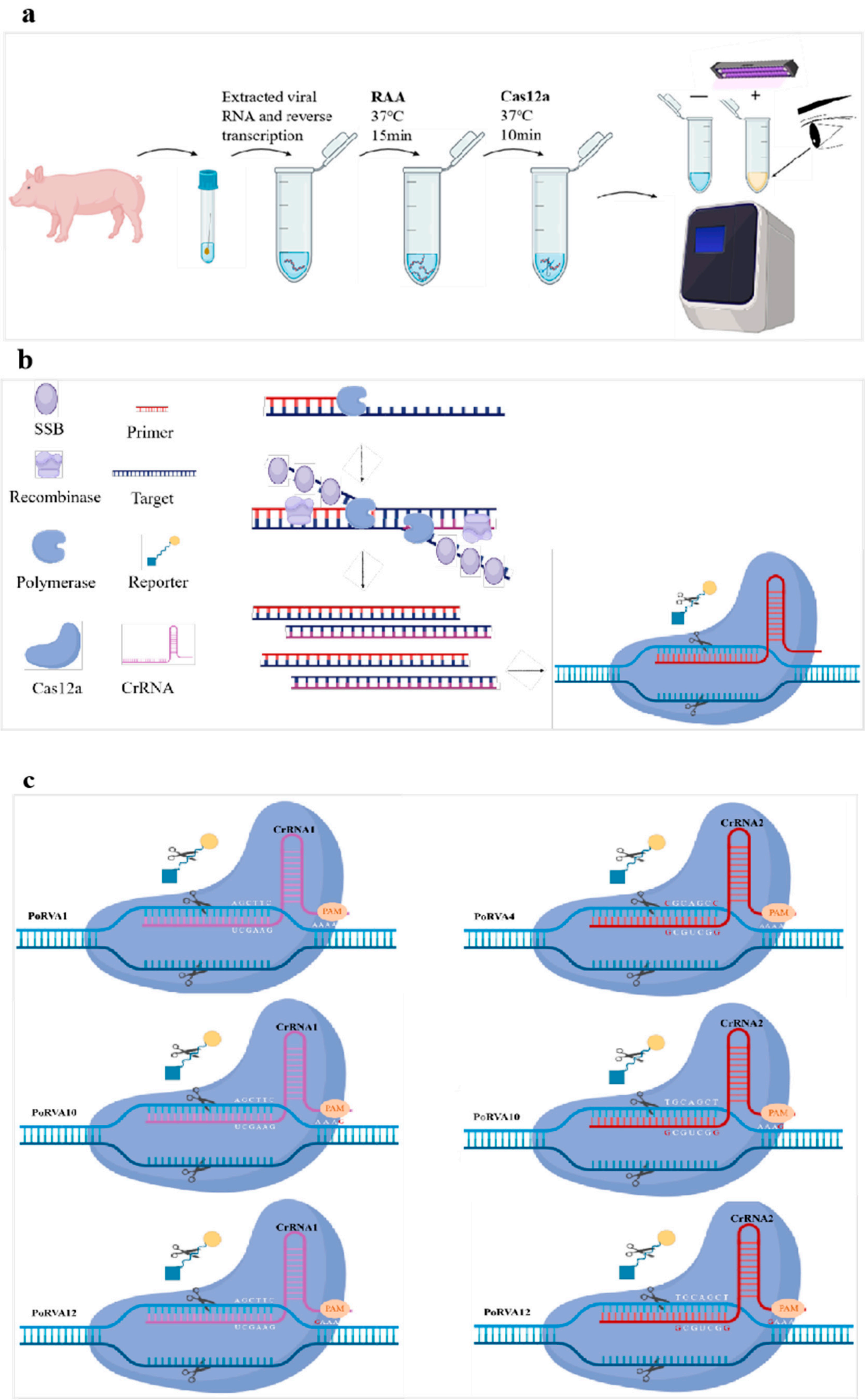

Supplementary Figure S1. The principle of RAA combining CRISPR/Cas12a For detecting porcine diarrhea-related viruses**. a** Principle of Cas12a for detecting G5 and G9 type PoRVA, **b** Detection Process Chart, **c** Principle of RAA and Cas12a.
